# Cross-Lineage Influenza B and Heterologous Influenza A Antibody Responses in Vaccinated Mice: Immunologic Interactions and B/Yamagata Dominance

**DOI:** 10.1371/journal.pone.0038929

**Published:** 2012-06-22

**Authors:** Danuta M. Skowronski, Marie-Eve Hamelin, Naveed Z. Janjua, Gaston De Serres, Jennifer L. Gardy, Chantal Rhéaume, Xavier Bouhy, Guy Boivin

**Affiliations:** 1 Communicable Disease Prevention and Control Services, British Columbia Centre for Disease Control, Vancouver, British Columbia, Canada; 2 School of Population and Public Health, University of British Columbia, Vancouver, British Columbia, Canada; 3 Department of Microbiology and Immunology, Laval University, Québec, Québec, Canada; 4 Department of Micrbiology and Immunology, Centre Hospitalier Universitaire de Québec, Québec, Québec, Canada; 5 Department of Social and Preventive Medicine, Faculty of Medicine, Laval University, Québec, Québec, Canada; 6 Direction of Biological and Occupational Risks, Institut national de santé publique du Québec, Québec, Canada; 7 Department of Microbiology and Immunology, University of British Columbia, Vancouver, British Columbia, Canada; The University of Adelaide, Australia

## Abstract

The annually reformulated trivalent inactivated influenza vaccine (TIV) includes both influenza A/subtypes (H3N2 and H1N1) but only one of two influenza B/lineages (Yamagata or Victoria). In a recent series of clinical trials to evaluate prime-boost response across influenza B/lineages, influenza-naïve infants and toddlers originally primed with two doses of 2008–09 B/Yamagata-containing TIV were assessed after two doses of B/Victoria-containing TIV administered in the subsequent 2009–10 and 2010–11 seasons. In these children, the Victoria-containing vaccines strongly recalled antibody to the initiating B/Yamagata antigen but induced only low B/Victoria antibody responses. To further evaluate this unexpected pattern of cross-lineage vaccine responses, we conducted additional immunogenicity assessment in mice. In the current study, mice were primed with two doses of 2008–09 Yamagata-containing TIV and subsequently boosted with two doses of 2010–11 Victoria-containing TIV (Group-Yam/Vic). With the same vaccines, we also assessed the reverse order of two-dose Victoria followed by two-dose Yamagata immunization (Group-Vic/Yam). The Group-Yam/Vic mice showed strong homologous responses to Yamagata antigen. However, as previously reported in children, subsequent doses of Victoria antigen substantially boosted Yamagata but induced only low antibody response to the immunizing Victoria component. The reverse order of Group-Vic/Yam mice also showed low homologous responses to Victoria but subsequent heterologous immunization with even a single dose of Yamagata antigen induced substantial boost response to both lineages. For influenza A/H3N2, homologous responses were comparably robust for the differing TIV variants and even a single follow-up dose of the heterologous strain, regardless of vaccine sequence, substantially boosted antibody to both strains. For H1N1, two doses of 2008–09 seasonal antigen significantly blunted response to two doses of the 2010–11 pandemic H1N1 antigen. Immunologic interactions between influenza viruses considered antigenically distant and in particular the cross-lineage influenza B and dominant Yamagata boost responses we have observed in both human and animal studies warrant further evaluation.

## Introduction

Since 1980, two lineages of influenza B viruses, represented by B/Yamagata/16/1988-like and B/Victoria/2/1987-like strains, have been recognized based on their antigenically distinct hemagglutinin (HA) surface proteins [1]. After an absence of more than ten years in North America, the Victoria lineage re-appeared in 2001 and at the end of 2002, a reassortment event occurred such that all type B viruses from 2003 onward bear the Yamagata neuraminidase (NA) [2]. Strains descended from both lineages variously contribute to annual influenza activity.

The annually reformulated trivalent inactivated influenza vaccine (TIV) contains both influenza A/subtypes (A/H3N2 and A/H1N1) but only one of the two major influenza B/lineages (Victoria or Yamagata). Young children are less likely to have had priming experience with influenza, and it is thus recommended that previously unvaccinated children <9 years of age receive two TIV doses for their initiating series, and a single dose annually thereafter [3]. This recommendation assumes effective prime-boost across related antigenic variants within a given influenza A/subtype, but does not account for major change in the influenza B/lineage from year-to-year.

In a recent series of clinical trials to assess prime-boost response across influenza B/lineages, we followed children enrolled as influenza-naïve infants and toddlers given two doses of the 2008–09 Vaxigrip split TIV (Sanofi Pasteur; Lyon, France) containing influenza B/Yamagata antigen [4]. The following year, a subset of these children was administered, per recommendation, a single dose of the 2009–10 Vaxigrip containing B/Victoria-lineage antigen [5]. A single dose of the 2009–10 Victoria antigen strongly recalled response to the 2008–09 priming Yamagata antigen, but titres to the immunizing Victoria antigen remained low. To assess whether another dose might recuperate a better Victoria response, a further subset was enrolled the next season to receive a single dose, per recommendation, of the same Victoria antigen in the 2010–11 Vaxigrip [3], [5]. That further dose, however, did not well improve the Victoria response, but again boosted titres to the Yamagata priming antigen.

It is unclear whether the cross-lineage influenza B results we observed in young children were specific to a particular product, antigen, or sequence of influenza B/lineage prime-boost. Few prior studies have specifically assessed cross-lineage influenza B vaccine responses [6]–[8] and additional opportunity to assess this has been limited to date by use of the same Victoria lineage antigen in the 2011–12 TIV. To further explore the unexpected cross-lineage influenza B responses we observed in naïve children, we conducted an animal study in which influenza-naïve mice were immunized with a different manufacturer’s TIV products from the same seasons, including the same Yamagata-Victoria sequence but also the reverse order of Victoria-Yamagata vaccine administration.

## Methods

### Ethics Statement

Animal procedures were approved by the Institutional Animal Care Committee at Laval University according to the guidelines of the Canadian Council on Animal Care.

### Mouse Immunization and Follow-Up

TIV immunogenicity was assessed in two groups of fifty 6–8-week-old female BALB/c mice (Charles River). All immunizations and serologic testing were conducted in blinded fashion. Animals were housed five per HEPA-filtered cage. Food and water were available *ad libitum*.

Mice belonging to Group-Yam/Vic received two immunizations with 2008–09 TIV (Yamagata lineage antigen) followed by two immunizations with 2010–11 TIV (Victoria lineage antigen). On day 0, these mice were immunized intramuscularly with 100 µl of a single lot of 2008–09 Fluviral non-adjuvanted split TIV (GlaxoSmithKline; Laval, Quebec, Canada) containing 3.0 µg HA [9] of each antigen i.e. B/Florida/4/2006(Yamagata)-like, A/Uruguay/716/2007(NYMC X-175C)(H3N2) (antigenically-equivalent to the WHO-recommended A/Brisbane/10/2007(H3N2) strain), and A/Brisbane/59/2007(H1N1)-like [10], [11]. A second immunization was repeated on day 14 with the same formulation. Two months later, another round of two 14-day-spaced immunizations was given in the same mice with a single lot of the 2010–11 Fluviral non-adjuvanted split TIV containing three different antigens: B/Brisbane/60/2008(Victoria)-like (a major influenza B lineage-level change compared with the 2008–09 component), A/Victoria/210/2009(NYMC X-187)(H3N2) (antigenically-equivalent to the WHO-recommended A/Perth/16/2009(H3N2) strain, itself a drift variant compared with the 2008–09 component), and A/California/7/2009(H1N1)-like (i.e. A/(H1N1)pdm09, a major H1N1 pandemic change compared with the 2008–09 seasonal component) [11], [12]. Supporting Information provides detail on the relatedness of 2008–09 and 2010–11 TIV and study antigens (Table S1, Table S2, Table S3, Table S4).

Group-Vic/Yam mice were immunized on the same dates in the same way using the same vaccine lots but in reverse sequence i.e. two immunizations with 2010–11 TIV (Victoria lineage antigen) followed by two immunizations with 2008–09 TIV (Yamagata lineage antigen).

All immunizations occurred between end-May and mid-August, 2011. Single radial immuno-diffusion (SRID) testing at >2 years post-expiry of the 2008–09 TIV lot that was used confirmed that all three strains still met standard potency requirements [Personal Communication, Dr. Rajesh Gupta, Center for Biologics Evaluation and Research, US Food and Drug Administration]. The 2010–11 TIV was administered within specified vaccine expiry.

Blood was collected prior to each immunization by cheek vein and two weeks after final immunization by cardiac puncture. Due to small blood volume, serum samples from the five mice per cage were pooled, rendering an effective sample size of 10 per group per time point.

### Serologic Assays

Sera were tested in duplicate for antibodies to non-inactivated viruses by hemagglutination inhibition (HI) assay for influenza A and B and additionally by microneutralization (MN) assay for influenza B according to methods described below. Test viruses were supplied by GlaxoSmithKline (GlaxoSmithKline; Laval, Quebec, Canada) with the exception of the A/California/7/2009(H1N1)-like virus, for which a Quebec isolate was used (GenBank accession numbers FN434457-FN434464). For the WHO-recommended A/Brisbane/10/2007(H3N2)-like vaccine component of the 2008–09 TIV, A/Uruguay/716/2007(H3N2) was used as the antigenically equivalent test strain (“Brisbane(H3N2)-like”). Titres <10 were assigned a value of 5. Where there was discordance in duplicate assay results, the lower value was recorded. Primary antibody endpoints included group geometric mean titres (GMTs), post- versus pre-immunization GMT ratio (GMTR) and proportion with titre ≥40.

#### Hemagglutination inhibition (HI) assay

HI assay was based on WHO protocol with modification as detailed below [13], [14]. Non-specific inhibitors were removed from serum by overnight treatment with receptor destroying enzyme (Denka Seiken, Tokyo, Japan). Physiologic saline solution was then added to achieve a 1∶10 dilution, followed by incubation with packed turkey red blood cells (TRBC) or guinea pig red blood cells (GPRBC) at 4°C for 60 min to remove non-specific agglutinins (Lampire Biological Laboratories Inc., Pipersville, PA). Treated serum was serially diluted in 25 µl of PBS and then mixed with an equal volume of PBS containing 4 hemagglutinin units of the different influenza A or B viruses. After 30 min of incubation at room temperature, 50 µl of 0.7% TRBC solution was added to the mixture then incubated for 30–60 min before evaluation of hemagglutination. For A/Brisbane/59/2007(H1N1) HI was also conducted with 1.2% GPRBC. The HI titer was recorded as the reciprocal of the last dilution that inhibited hemagglutination.

#### Microneutralization

Sera were first inactivated for 30 min at 56°C. Beginning with a 1∶10 dilution two-fold serial dilutions of sera were mixed with equal volume of medium (Dulbecco’s Modification of Eagle’s Medium with L-glutamine, 4.5 g/L glucose and sodium pyruvate) containing 100 TCID50 of influenza B viruses. After a 2-h incubation at 37°C in 5% CO_2_ humidified atmosphere, the residual infectivity of the virus-serum mixture (50 µl) was determined by infecting confluent MDCK cells. Neutralizing antibody titers were defined as the reciprocal of the highest dilution of serum that completely neutralized the infectivity of the virus as determined by the absence of cytopathic effect at day 4 post-infection [15].

### Gene Sequencing

To assist in the interpretation of immunogenicity findings, surface protein sequences of influenza B study viruses were compared against representative B/Yamagata/16/1988 and B/Victoria/2/1987 lineage viruses; relevant influenza A and B vaccine strains for comparison were also downloaded from NCBI’s Influenza Virus Resource [16]. Antigenic regions for influenza B have not been defined, therefore pairwise identities were calculated over the HA1 domain of hemagglutinin and the full-length neuraminidase for both influenza A and B vaccine and study strains. Pairwise identities were calculated from alignments generated with MAFFT [17].

## Results

### Influenza B

Group-Yam/Vic animals confirmed the previous pattern of influenza B responses we first reported in children (Table 1) [4], [5]. The Yamagata antigen induced strong antibody response to the homologous virus, but little cross-reactive antibody to the Victoria antigen. A single follow-up dose with Victoria antigen substantially boosted antibody to the priming Yamagata lineage but titres to the immunizing Victoria antigen remained significantly lower even with two doses.

**Table 1:** Cross-lineage influenza B antibody responses measured by HI and MN assays.*

GROUP Yam/Vic: 2008–09/2010–11 TIV Sequence			Initiating antigen					Boosting antigen			
Test antigen			Yamagata			Yamagata		Victoria		Victoria	
Yamagata	Pre- vaccination		Post 2008–09 TIV 1^a^			Post 2008–09 TIV 2^b^		Post 2010–11 TIV 1 a		Post 2010–11 TIV 2^a^	
	HI	MN	HI	MN		HI	MN	HI	MN	HI	MN
GMT (95%CI)	5.7(4.7–7.1)	7.6(5.9–9.8)	40.0(26.7–60)	28.3(21.8–36.7)		242.5(187.7–313.3)	98.5(77.5–125.1)	1940.1(1277.1–2947.3)	394(281.9–550.6)	1940.1(1501.8–2506.3)	640(506.6–808.5)
GMTR†			7.02	3.72		42.54	12.96	8.00	4.00	1.00	1.62
% titre ≥40 (95%CI)	0	0	70 (35 – 100)	50 (12 – 88)		100	100	100	100	100	100
**Victoria**											
GMT (95%CI)	5.0	5.4 (4.6–6.3)	5.0	11.5 (9.3–14.2)		5.0	23 (18.6–28.3)	20.0 (12.5–31.9)	30.3 (21.4–42.9)	17.4 (8.4–36.2)	34.8 (25.4–47.6)
GMTR†			1.00	2.13		1.00	4.26	4.00	1.32	0.87	1.15
% titre ≥40 (95%CI)	0	0	0	0		0	20 (0 – 50)	20 (0 – 50)	50 (12 – 88)	40 (3 – 77)	70 (35–100)
**GROUP Vic/Yam: 2010–11/2008–09 TIV Sequence**			**Initiating antigen**					**Boosting antigen**			
**Test antigen**			**Victoria**		**Victoria**			**Yamagata**		**Yamagata**	
**Yamagata**	**Pre- vaccination**		**Post 2010–11 TIV 1** a		**Post 2010–11 TIV 2** b			**Post 2008–09 TIV 1** a		**Post 2008–09 TIV 2** a	
	**HI**	**MN**	**HI**	**MN**	**HI**		**MN**	**HI**	**MN**	**HI**	**MN**
GMT (95%CI)	6.2(4.8–7.8)	6.2(4.8–7.8)	8.1(5.8–11.4)	14.1(10.9–18.4)	24.6(15.4–39.4)		26.4(20.4–34.1)	844.5(495.6–1439.1)	278.6(203.6–381.2)	970.1(638.6–1473.6)	320(229.9–445.4)
GMTR†			1.31	2.27	3.97		4.26	34.33	10.55	1.15	1.15
% titre ≥40 (95%CI)	0	0	0	0	40 (3 – 77)		40 (3 – 77)	100	100	100	100
**Victoria**											
GMT (95%CI)	5.0	5.0	5.0	12.3 (9.7–15.6)	30.3 (23.5–39.2)		42.9 (32.4–56.8)	211.1 (149.3–298.6)	160 (126.7–202.1)	259.9 (186–363.2)	278.6 (203.6–381.2)
GMTR†			1.00	2.46	6.06		8.58	6.97	3.73	1.23	1.74
% titre ≥40 (95%CI)	0	0	0	0	60 (23 – 97)		90 (67 – 100)	100	100	100	100

HI  =  hemagglutination inhibition; MN  =  microneutralization; TIV  =  trivalent inactivated influenza vaccine. Note that undetectable titres <10 were assigned a value of 5.

* Unit of analysis pooled sera from 5 mice, thus 10 pools from 50 mice were available for this experiment.

a Measured two weeks after specified TIV dose;

b Measured two months after specified TIV dose.

† Compared to pre-immunization for initiating antigens; compared to immediately preceding titre for boosting antigens.

Yamagata  =  Florida/4/06(Yamagata)-like, the Yamagata lineage antigen included in the northern hemisphere 2008–09 TIV.

Victoria  =  Brisbane/60/08(Victoria)-like, the Victoria lineage antigen included in the northern hemisphere 2009–10 and 2010–11 TIV.

Group-Vic/Yam animals provide additional insight not available in the earlier pediatric trials (Table 1) [4], [5]. After two initiating doses, the homologous response to the Victoria antigen in the Vic/Yam animals was low. Follow-up immunization with Yamagata antigen, however, revealed the Victoria antigen to have nevertheless been an effective priming immunogen, even cross-lineage: with a single dose of Yamagata antigen, titres were significantly raised to both lineages. Titres to Yamagata significantly exceeded those following two homologous priming Yamagata doses in Group-Yam/Vic mice and Victoria titres were also significantly raised 4-7-fold.

Overall, there was dominant Yamagata boost response regardless of the lineage used for the initiating series or subsequent boosting. The same patterns were observed by HI and MN, but were more pronounced by HI (Table 1).

### Influenza A

For H3N2, homologous responses were comparably robust for the 2008–09 and 2010–11 TIV antigens (Table 2). Cross-reactive heterotypic responses were low. However, even a single follow-up dose of the heterologous strain, regardless of the sequence of vaccine receipt, elevated the antibody response to both strains.

**Table 2:** Cross-strain influenza A(H3N2) antibody responses measured by HI assay.*

GROUP Yam/Vic: 2008–09/2010–11 TIV Sequence		Initiating antigen		Boosting antigen	
Test Antigen		Brisbane-like	Brisbane-like	Perth	Perth
Brisbane-like	Pre- vaccination	Post 2008–09 TIV 1^a^	Post 2008–09 TIV 2^b^	Post 2010–11 TIV 1^a^	Post 2010–11 TIV 2^a^
GMT (95%CI)	5.0	32.5 (25.6–41.3)	130 (102.3–165.1)	1194.3 (729.4–1955.5)	1114.3 (667.7–1859.6)
GMTR†		6.50	26.0	9.19	0.93
% titre ≥40 (95%CI)	0	70 (35–100)	100	100	100
**Perth**					
GMT (95%CI)	5.0	5.0	6.2 (4.4–8.6)	557.2 (302.9–1024.9)	519.8 (324.8–832.1)
GMTR†		1	1.24	89.87	0.93
% titre ≥40 (95%CI)	0	0	0	100	100
**GROUP Vic/Yam: 2010–11/2008–09 TIV Sequence**		**Initiating antigen**		**Boosting antigen**	
**Test antigen**		**Perth**	**Perth**	**Brisbane-like**	**Brisbane-like**
**Brisbane-like**	**Pre- vaccination**	**Post 2010–11 TIV 1** a	**Post 2010–11 TIV 2** b	**Post 2008–09 TIV 1** a	**Post 2008–09 TIV 2** a
GMT (95%CI)	5.0	5.0	6.2 (4.8–7.8)	343 (189.4–621)	452.5 (296.9–689.7)
GMTR†		1.00	1.24	55.32	1.32
% titre ≥40 (95%CI)	0	0	0	100	100
**Perth**					
GMT (95%CI)	5.0	11.5 (6.5–20.2)	183.8 (134.3–251.5)	787.9 (523.8–1185.2)	844.5 (597.1–1194.4)
GMTR†		2.3	36.76	4.29	1.07
% titre ≥40 (95%CI)	0	20 (0 – 50)	100	100	100

HI = hemagglutination inhibition; TIV  =  trivalent inactivated influenza vaccine. Note that undetectable titres <10 were assigned a value of 5.

* Unit of analysis pooled sera from 5 mice, thus 10 pools from 50 mice were available for this experiment.

a Measured two weeks after specified TIV dose;

b Measured two months after specified TIV dose.

† Compared to pre-immunization for priming antigens; compared to immediately preceding titre for boosting antigens.

Brisbane-like  =  A/Uruguay/716/2007(NYMC 175C)(H3N2) considered antigenically equivalent to the WHO recommended A/Brisbane/10/2007(H3N2) component of the 2008–09 northern hemisphere TIV.

Perth  =  A/Perth/16/2009(H3N2)-like, component of the northern hemisphere 2010–11 TIV.

For H1N1 (Table 3), the 2008–09 A/Brisbane/59/2007 antigen induced little antibody response; the same antigen was also cited in this manufacturer’s product monograph for its failure to meet immunogenicity criteria among older adults in pre-season trials conducted for 2009–10 [18]. Repeat HI assay using GPRBC did not change these findings in mice. Despite this, response to two-dose A/California/07/2009(H1N1) immunization was significantly blunted when preceded by A/Brisbane/59/2007 priming compared to two-dose A/California/07/2009(H1N1) immunization of naïve animals.

**Table 3:** Cross-strain influenza A(H1N1) antibody responses measured by HI assay.*

GROUP Yam/Vic: 2008–09/2010–11 TIV Sequence		Initiating antigen		Boosting antigen	
Test Antigen		Brisbane	Brisbane	California	California
Brisbane	Pre- vaccination	Post 2008–09 TIV 1^a^	Post 2008–09 TIV 2^b^	Post 2010–11 TIV 1^a^	Post 2010–11 TIV 2^a^
GMT (95%CI)	5.0	5.0	10 (6.7–15)	13.2 (9.3–18.7)	8.1 (5.8–11.4)
GMTR†		1.00	2.00	1.32	0.61
% titre ≥40 (95%CI)	0	0	0	10 (0–33)	0
**California**					
GMT (95%CI)	5.0	5.0	5.0	5.0	16.2 (8–32.8)
GMTR†		1.00	1.00	1.00	3.24
% titre ≥40 (95%CI)	0	0	0	0	30 (0–65)
**GROUP Vic/Yam: 2010–11/2008–09 TIV Sequence**		**Inititating antigen**		**Boosting antigen**	
**Test antigen**		**California**	**California**	**Brisbane**	**Brisbane**
**Brisbane**	**Pre- vaccination**	**Post 2010–11 TIV 1** a	**Post 2010–11 TIV 2** b	**Post 2008–09 TIV 1** a	**Post 2008–09 TIV 2** a
GMT (95%CI)	5.0	5.0	5.0	5.0	5.4 (4.6–6.3)
GMTR†		1.00	1.00	1.00	1.08
% titre ≥40 (95%CI)	0	0	0	0	0
**California**					
GMT (95%CI)	5.0	5.7 (4.7–7.1)	74.6 (56.3–98.9)	74.6 (63.8–87.3)	45.9 (33.6–62.9)
GMTR†		1.14	14.92	1.00	0.62
% titre ≥40 (95%CI)	0	0	100	100	90 (67–100)

HI  =  hemagglutination inhibition; TIV  =  trivalent inactivated influenza vaccine. Note that undetectable titres <10 were assigned a value of 5.

* Unit of analysis pooled sera from 5 mice, thus 10 pools from 50 mice were available for this experiment.

a Measured two weeks after specified TIV dose;

b Measured two months after specified TIV dose.

† Compared to pre-immunization for initiating antigens; compared to immediately preceding titre for boosting antigens.

Brisbane  =  A/Brisbane/59/2007(H1N1)-like, component of the northern hemisphere 2008–09 and 2009–10 TIV.

California  =  A/California/07/2009(H1N1)-like, component of the northern hemisphere 2010–11 TIV.

### Gene Sequencing

The Yamagata/16/1988 and Victoria/2/1987 reference strains showed 92.8% cross-lineage pairwise identities in their HA1, whereas descendant strains showed reduced cross-lineage pairwise identity (average 90.2% ) (Table S1). Although these cross-lineage identities are less than the pairwise identities observed between variants within an influenza B/lineage or A/H3N2 subtype (≥96%), they are greater than the pairwise identities across the most recent seasonal and pandemic H1N1 strains (∼72%) and far exceed the pairwise identity across influenza A/H3 and A/H1 subtypes (∼35%) (Tables S1, Table S3).

NA identities were more conserved across influenza B/lineages (Table S2). Despite 2002 reassortment, identity was greater between Yamagata/16/1988 and Victoria/2/1987 reference strains (96.8%) than cross-lineage between their most recent descendants (95.5%), suggesting possible drift in the NA. Influenza B cross-lineage identities for NA appear greater than the pairwise identities observed across the most recent seasonal and pandemic N1 strains (∼81%) and also far exceed the pairwise identity across the most recent influenza A/N2 and A/N1 subtype strains (∼42%) (Tables S2, S4).

## Discussion

Influenza B contributes significantly to winter respiratory illness, especially among children, causing seasonal epidemics every 2–4 years [19]. Given recognized variability in vaccine protection and proposals for a revised quadrivalent formulation, better understanding of influenza B vaccine responses and possible immunologic interactions is important [19].

In the current study, we confirmed dominant Yamagata responses reported for the first time during a recent series of clinical trials in which naïve children were initiated per recommendation with two doses of Yamagata antigen and boosted with Victoria antigen [4], [5]. Here we observed the same effect in naïve adult mice using another manufacturer’s TIV products from the same seasons. To assess the directionality of this pattern of cross-lineage prime-boost responses, we additionally included mice that were instead initiated with Victoria and subsequently boosted with Yamagata lineage antigen. This showed that regardless of priming lineage, the boost response to Yamagata was persistently dominant. The Victoria antigen induced weaker homologous response but was still an effective immunogen for single-dose cross-lineage Yamagata boost. Conversely, the Yamagata antigen induced robust homologous prime-boost responses, but subsequent response to Victoria antigen remained low.

Although the antigenic distance between influenza B/lineages has generally been compared to that across influenza A/subtypes [19], our HA1 sequence analysis showed almost three-fold higher overall pairwise identity between B/Yamagata and B/Victoria than across A/H3 and A/H1 subtypes (Tables S1 and S3). These may be lower in specific antibody-binding sites, but cannot be determined without influenza B-specific HA epitope maps. Our immunogenicity findings also suggest some degree of cross-lineage immune recognition, since B/Victoria antigen both effectively primed for Yamagata and substantially recalled priming Yamagata responses. In an earlier study, Levandowski also showed this cross-lineage immune recognition, with two-dose Yamagata immunization inducing substantial Yamagata responses and recalling Victoria responses in previously Victoria-primed but not unprimed children [6]. As in the current mouse study and using the same 2010–11 TIV, Gilca et al have also recently reported reduced immunogenicity for the B/Victoria antigen in young Quebec children previously primed with Yamagata antigen [20]. Similarly, among Canadian children 12–59 months of age immunized with the 2009–10 adjuvanted pandemic H1N1 vaccine, Langley et al have reported significantly lower 2010–11 B/Victoria (but not lower influenza A) responses in those who had also previously received the 2009–10 TIV [21]. Although the same B/Victoria antigen was retained across the 2009–10 and 2010–11 TIV, this paper did not report immunization history prior to 2009: it is therefore unclear to what extent the possibly correlated receipt of 2008–09 or earlier Yamagata-containing TIV may have also been influential [21]. Among adults, the B/Victoria antigen has also been reported as significantly less immunogenic than influenza A components for both the 2009–10 and 2010–11 TIV but analysis based on prime-boost history is yet more complex and challenging for adults and was not presented in these publications [22], [23].

The underlying immunologic mechanism for the Yamagata dominance we report, if real, warrants better understanding. Such one-way cross-lineage cross-reactivity has also been highlighted by the World Health Organization [14], although reasons for this have not been elaborated. In the current study, Group-Yam/Vic mice might be consistent with original antigenic sin, whereby antibody to priming antigens is preferentially recalled at the expense of response to subsequent related-but-distinct viruses [24]–[26]. However, the Group-Vic/Yam mice suggest this imprinting does not apply in reverse: Yamagata boost was able to overcome the Victoria priming experience. For influenza A, intravirionic antigenic competition is thought to exist between HA and NA proteins recognized as an assembly by cells of the immune system [27]. HA immune-dominance is thought to suppress NA responses under most conditions. However, in a process termed “reverse antigen competition”, the relative importance of the NA immune response is increased, with anti-NA suppressing HA response in NA-primed populations lacking HA cross-reactive antibodies [27]. Closer cross-lineage homology for NA might signal its more prominent role and account for the Yamagata antigen’s dominance among Group-Yam/Vic responses when boosted with Victoria HA. A greater role for anti-NA antibody may also explain diversity in the NA protein. This hypothesis, however, is weakened by uncertainty in the NA content of TIV, the uncertain role of NA-antibody in influencing HI results and the greater difficulty in reconciling with the Group-Vic/Yam findings. We did not assess anti-NA responses but these may also be of follow-up interest. Neither original antigenic sin nor reverse antigenic dominance have been specifically studied for influenza B. In fact, studies of influenza B immune responses are lacking generally. Other cross-lineage interactions, overriding epitope-specific effects, immunologic tolerance, suppression or other mechanisms may be involved [28]. Whatever the mechanism invoked, it must be able to account for the hierarchical responses we observed based on the sequence of administration for some but not all antigens, heterotypic in one direction but not the other. Similarly, the mechanism for diminished A/(H1N1)pdm09 responses when preceded by antigenically distant seasonal H1N1 priming is unclear. Blunting of A/(H1N1)pdm09 vaccine-induced antibody response in association with prior seasonal vaccine receipt has also been reported in human immunogenicity trials [29]–[34], shown here also in mice despite absence of seasonal H1 antibody induction. Conversely, pandemic H1N1 immunization has been observed to boost pre-existing heterosubtypic neutralizing antibody levels [35]. Our study further exposes possible immunologic interactions between related but antigenically distant influenza viruses, including cross-lineage influenza B effects, but it cannot elucidate the mechanisms. Further evaluation is needed.

Limitations of this study warrant consideration. It is recognized that mice do not necessarily represent human immune responses in quantity, quality or kinetics. In the current study, spacing between prime-boost vaccine doses was two weeks and between seasonal formulations was two months. This was intentionally shortened from the four-week prime-boost interval and annual administration recommended for children to reflect the shortened lifespan and time scale of mice relative to humans. The published literature references a range of two-dose influenza vaccine spacing in mice including the use of 2[36]–[39], 3[37], [40]–[42] or 4[43]–[45] week intervals. Since in this study we used the same schedule regardless of antigen or its order of administration, the chosen interval between prime-boost doses while potentially influential is unlikely to explain our differential findings on that basis. Although the 2008–09 TIV was nearly 2 years expired when used, we elicited excellent Yamagata and H3N2 responses; reduced H1N1 responses were in keeping with human immunogenicity findings also reported with that manufacturer’s antigen [18]. Furthermore, standard SRID testing confirmed ongoing potency of the vaccine lot that was used. Differences in timing between the first (2 month) versus final (2 week) two-dose blood draw for the initiating versus boosting series should be taken into account–2 weeks should be sufficient for boost response but the possibility that final antibody rise had not yet been achieved or conversely that waning had occurred over the 2 month interval cannot be ruled out. Again, this is unlikely to explain differential findings by antigen since the same schedule of blood draw was followed for each immunization series. To ensure sufficient sera for assessment across multiple assays and antigens, we pooled from several mice, potentially masking underlying variability. Differences in laboratory protocols and a degree of error in antibody assays are acknowledged [46], [47]. We did not include ether-treatment of the influenza B antigens for HI assay, an approach thought to increase sensitivity but decrease specificity of influenza B HI assay results [48], [49]. However, ether treatment did not alter the overall pattern observed in the earlier pediatric study [5] and trends were consistent by HI and MN in both studies. We emphasize observed trends over absolute values. We cannot rule out that our observations are particular to the specific influenza B strains used in both the pediatric and mice studies from the same seasons. Studies across additional seasons, strains and vaccine formulations (live, inactivated, trivalent, quadrivalent, adjuvanted etc) would be worthwhile. Ultimately, mouse studies are supportive but cannot alone be interpreted as conclusive for human immune responses [50]. The main strength of our findings, however, is the consistency across human and animal studies, across manufacturer’s TIV products, across HI and MN assays, and in this study under controlled experimental conditions, suggesting the interactions we report are unlikely to have been due to bias or chance alone. In that regard, they warrant further detailed evaluation.

In conclusion, we highlight dominant Yamagata responses previously shown for the first time in naïve children [4], [5], and here confirmed in mice regardless of the initiating lineage of influenza B priming. If further confirmed, these findings have both scientific and practical implications for influenza vaccine protection, trivalent or quadrivalent, in young children. Further study across additional seasons, antigens and products, including trivalent and quadrivalent formulations, as well as epitope-specific and mechanistic investigations will be important to clarify the nature and relevance of these immunologic interactions.

## Supporting Information

Table S1 **Pairwise identity (% (number of mutations)) in influenza B hemagglutinin 1 (HA1) peptide (Amino acids 18–361).**
(DOC)

Table S2 **Pairwise identity (% (number of mutations)) in influenza B neuraminidase (NA) protein (Amino acids 1–466).**
(DOC)

Table S3 **Pairwise identity (% (number of mutations)) in influenza A hemagglutinin 1 (HA1) peptide (Amino acids 17/18–∼345).**
(DOC)

Table S4 **Pairwise identity (% (number of mutations)) in influenza A neuraminidase (NA) protein (Amino acids 1–469).**
(DOC)
