# Supplementary material for: Cross-Lineage Influenza B and Heterologous Influenza A Antibody Responses in Vaccinated Mice: Immunologic Interactions and B/Yamagata Dominance
Source: PLoS One. 2012 Jun 22;7(6):e38929. doi: 10.1371/journal.pone.0038929 (PMC3382187; doi:10.1371/journal.pone.0038929)
Supplement: Table S1 — Pairwise identity (% (number of mutations)) in influenza B hemagglutinin 1 (HA1) peptide (Amino acids 18–361). (DOC) [file pone.0038929.s001.doc]

**Table S1. Pairwise identity (% (number of mutations)) in influenza B hemagglutinin 1 (HA1) peptide**

**(Amino acids 18**-361)

|  | **B/Victoria/2/1987*** | **B/HongKong/330/2001*** | **B/Malaysia/2506/2004*** | **B/Brisbane/60/2008*** | **Study Brisbane/60/2008*** | **B/Yamagata/16/1988**** | **B/Beijing/184/1993**** | **B/Sichuan/379/1999**** | **B/Shanghai/361/2002**** | **B/Florida/4/2006**** |
| --- | --- | --- | --- | --- | --- | --- | --- | --- | --- | --- |
|  |  |  |  |  |  |  |  |  |  |  |
| **B/HongKong/330/2001*** | 95.9 (14) | – | – | – | – | – | – | – | – | – |
| **B/Malaysia/2506/2004*** | 95.7 (15) | 97.7 (8) | – | – | – | – | – | – | – | – |
| **B/Brisbane/60/2008*** | 95.7 (15) | 96.8 (11) | 98.6 (5) | – | – | – | – | – | – | – |
| **Study Brisbane/60/2008*** | 95.9 (14) | 97.1 (10) | 98.3 (6) | 99.7 (1) | – | – | – | – | – | – |
| **B/Yamagata/16/1988**** | 92.8 (25) | 91.9 (28) | 91.3 (30) | 91.6 (29) | 91.6 (29) | – | – | – | – | – |
| **B/Beijing/184/1993**** | 91.6 (29) | 91.0 (31) | 91.3 (30) | 91.0 (31) | 90.7 (32) | 96.8 (11) | – | – | – | – |
| **B/Sichuan/379/1999**** | 90.7 (32) | 89.9 (35) | 89.3 (37) | 89.6 (36) | 89.6 (36) | 94.2 (20) | 96.2 (13) | – | – | – |
| **B/Shanghai/361/2002**** | 91.0 (31) | 90.4 (33) | 90.1 (34) | 90.4 (33) | 90.4 (33) | 94.8 (18) | 96.8 (11) | 95.1 (17) | – | – |
| **B/Florida/4/2006**** | 91.0 (31) | 89.9 (35) | 89.6 (36) | 89.9 (35) | 89.9 (35) | 94.5 (19) | 96.2 (13) | 95.1 (17) | 98.3 (6) | – |
| **Study Florida/4/2006**** | 90.4 (33) | 89.9 (35) | 89.9 (35) | 90.1 (34) | 89.9 (35) | 94.8 (18) | 96.2 (13) | 93.9 (21) | 97.7 (8) | 98.5 (5) |

* Influenza B/Victoria lineage

** Influenza B/Yamagata lineage

TIV= trivalent inactivated influenza vaccine

**Note:**

B/Brisbane/60/2008 included in this study as the 2010-11 northern hemisphere TIV B/Victoria lineage component. Study Brisbane/60/2008-like had one mutation relative to reference strain (N212S).

B/Florida/4/2006 included in this study as the 2008-09 northern hemisphere TIV B/Yamagata lineage component. Study Florida/4/2006-like had five mutations relative to reference strain (K103R, S165I, N180Y, D211N, S244D).

Pairwise identities were calculated from alignments generated with MAFFT (Katoh K, Asimenos G, Toh H (2009). Multiple alignment of DNA sequences with MAFFT. Methods Mol Biol 537:39-64).
