# Supplementary material for: Cross-Lineage Influenza B and Heterologous Influenza A Antibody Responses in Vaccinated Mice: Immunologic Interactions and B/Yamagata Dominance
Source: PLoS One. 2012 Jun 22;7(6):e38929. doi: 10.1371/journal.pone.0038929 (PMC3382187; doi:10.1371/journal.pone.0038929)
Supplement: Table S2 — Pairwise identity (% (number of mutations)) in influenza B neuraminidase (NA) protein (Amino acids 1–466). (DOC) [file pone.0038929.s002.doc]

**Table S2. Pairwise identity (% (number of mutations)) in influenza B neuraminidase (NA) protein**

**(Amino acids 1 – 466)**

|  | **B/Victoria/2/1987*** | **B/HongKong/330/2001*** | **B/Malaysia/2506/2004*** | **B/Brisbane/60/2008*** | **Study Brisbane/60/2008*** | **B/Yamagata/16/1988**** | **B/Beijing/184/1993**** | **B/Sichuan/379/1999**** | **B/Shanghai/361/2002**** | **B/Florida/4/2006**** |
| --- | --- | --- | --- | --- | --- | --- | --- | --- | --- | --- |
|  |  |  |  |  |  |  |  |  |  |  |
| **B/HongKong/330/2001*** | 97.4 (12) | – | – | – | – | – | – | – | – | – |
| **B/Malaysia/2506/2004*** | 92.9 (33) | 92.7 (34) | – | – | – | – | – | – | – | – |
| **B/Brisbane/60/2008*** | 93.1 (32) | 92.1 (37) | 97.2 (13) | – | – | – | – | – | – | – |
| **Study Brisbane/60/2008*** | 92.2 (33) | 91.8 (35) | 97.4 (11) | 99.8 (1) | – | – | – | – | – | – |
| **B/Yamagata/16/1988**** | 96.8 (15) | 95.3 (22) | 94.4 (26) | 94.6 (25) | 93.9 (26) | – | – | – | – | – |
| **B/Beijing/184/1993**** | 95.9 (19) | 94.6 (25) | 95.7 (20) | 95.5 (21) | 95.3 (20) | 97.9 (10) | – | – | – | – |
| **B/Sichuan/379/1999**** | 94.6 (25) | 94.6 (25) | 97.6 (11) | 97.0 (14) | 96.9 (13) | 96.1 (18) | 97.4 (12) | – | – | – |
| **B/Shanghai/361/2002**** | 94.4 (26) | 94.0 (28) | 97.6 (11) | 96.8 (15) | 96.7 (14) | 95.7 (20) | 97.0 (14) | 99.1 (4) | – | – |
| **B/Florida/4/2006**** | 93.1 (32) | 93.1 (32) | 95.7 (20) | 95.5 (21) | 95.3 (20) | 94.8 (24) | 95.9 (19) | 97.4 (12) | 97.2 (13) | – |
| **Study Florida/4/2006**** | 93.3 (28) | 93.5 (27) | 95.7 (18) | 95.0 (21) | 95.2 (20) | 95.0 (21) | 96.2 (16) | 97.4 (11) | 97.1 (12) | 98.6 (6) |

* Influenza B/Victoria lineage

** Influenza B/Yamagata lineage

TIV= trivalent inactivated influenza vaccine

**Note:**

B/Brisbane/60/2008 included in this study as the 2010-11 northern hemisphere TIV B/Victoria lineage component. The NA of Study Brisbane/60/2008-like was sequenced over residues 18-442 only and had one mutation relative to the reference strain (K404E).

B/Florida/4/2006 included in this study as the 2008-09 northern hemisphere TIV B/Yamagata lineage component. The NA of Study Florida/4/2006-like was sequenced over residues 24-441 only and had six mutations relative to the reference strain (Q42R, A68T, T125K, K160E, K186R, D340N).

Pairwise identities were calculated from alignments generated with MAFFT (Katoh K, Asimenos G, Toh H (2009). Multiple alignment of DNA sequences with MAFFT. Methods Mol Biol 537:39-64).
