# Supplementary material for: Cross-Lineage Influenza B and Heterologous Influenza A Antibody Responses in Vaccinated Mice: Immunologic Interactions and B/Yamagata Dominance
Source: PLoS One. 2012 Jun 22;7(6):e38929. doi: 10.1371/journal.pone.0038929 (PMC3382187; doi:10.1371/journal.pone.0038929)
Supplement: Table S3 — Pairwise identity (% (number of mutations)) in influenza A hemagglutinin 1 (HA1) peptide (Amino acids 17/18–∼345). (DOC) [file pone.0038929.s003.doc]

**Table S3. Pairwise identity (% (number of mutations)) in influenza A hemagglutinin 1 (HA1) peptide**

**(Amino acids 17/18-~345)**

|  | **A/Perth/16/2009*** | **A/Victoria/201/2009 X-187*** | **Study Perth/16/2009*** | **A/Brisbane/10/2007*** | **A/Uruguay/716/2007 X-175C*** | **Study Uruguay/716/2007*** | **A/Brisbane/59/2007**** | **Study Brisbane/59/2007**** | **A/California/07/2009**** |
| --- | --- | --- | --- | --- | --- | --- | --- | --- | --- |
|  |  |  |  |  |  |  |  |  |  |
| **A/Victoria/201/2009 X-187*** | 98.2 (6) | – | – | – | – | – | – | – | – |
| **Study Perth/16/2009*** | 99.6 (1) | 97.7 (6) | – | – | – | – | – | – | – |
| **A/Brisbane/10/2007*** | 98.2 (6) | 97.0 (10) | 97.7 (6) | – | – | – | – | – | – |
| **A/Uruguay/716/2007 X-175C*** | 97.3 (9) | 96.0 (13) | 96.6 (9) | 99.1 (3) | – | – | – | – | – |
| **Study Uruguay/716/2007*** | 97.4 (8) | 96.1 (12) | 97.0 (8) | 99.4 (2) | 99.7 (1) | – | – | – | – |
| **A/Brisbane/59/2007**** | 35.0 (221) | 35.3 (220) | 37.8 (166) | 34.7 (222) | 34.7 (222) | 35.9 (202) | – | – | – |
| **Study Brisbane/59/2007**** | 36.5 (200) | 36.8 (199) | 38.2 (165) | 36.2 (201) | 36.2 (201) | 36.2 (201) | 99.4 (2) | – | – |
| **A/California/07/2009**** | 34.7 (222) | 34.7 (222) | 38.6 (164) | 34.7 (222) | 34.1 (224) | 35.6 (203) | 71.9 (92) | 71.5 (90) | – |
| **Study California/07/2009**** | 36.8 (208) | 36.8 (208) | 38.6 (164) | 36.5 (209) | 35.9 (211) | 35.6 (203) | 72.8 (89) | 72.4 (87) | 99.7 (1) |

* Influenza A/H3N2 subtype

** Influenza A/H1N1 subtype

TIV = trivalent inactivated influenza vaccine

**Note:**

A/Uruguay/716/2007 (NYMC X-175C) was the 2008-09 study TIV component considered antigenically-equivalent to the 2008-09 WHO-recommended H3N2 vaccine strain, A/Brisbane/10/2007. The HA of Study Uruguay/716/2007-like was sequenced over 310 residues of the HA1 peptide and had two mutations relative to A/Brisbane/10/2007(A154S, L210P).

A/Victoria/201/2009 (NYMC X-187) was the 2010-11 study TIV component considered antigenically-equivalent to the 2010-11 WHO recommended H3N2 vaccine strain, A/Perth/16/2009. The HA of Study Perth/16/2009-like was sequenced over 264 residues of the HA1 peptide and had one mutation relative to A/Perth/16/2009 (H199L).

A/Brisbane/59/2007 was the 2008-09 study TIV H1N1 component as recommended by the WHO, and used as the 2008-09 test antigen in this study. The HA of Study Brisbane/59/2007-like was sequenced over 314 residues of the HA1 peptide and had two mutations relative to A/Brisbane/59/2007 (N203D, F277L).

A/California/07/2009 was the 2010-11 study TIV H1N1 component as recommended by the WHO, and used as the 2010-11 test antigen in this study. The HA of Study California/07/2009-like was sequenced over the complete HA1 peptide and contained one mutation relative to A/California/07/2009 (S200P)

Pairwise identities were calculated from alignments generated with MAFFT (Katoh K, Asimenos G, Toh H (2009). Multiple alignment of DNA sequences with MAFFT. Methods Mol Biol 537:39-64).
