# Supplementary material for: Cross-Lineage Influenza B and Heterologous Influenza A Antibody Responses in Vaccinated Mice: Immunologic Interactions and B/Yamagata Dominance
Source: PLoS One. 2012 Jun 22;7(6):e38929. doi: 10.1371/journal.pone.0038929 (PMC3382187; doi:10.1371/journal.pone.0038929)
Supplement: Table S4 — Pairwise identity (% (number of mutations)) in influenza A neuraminidase (NA) protein (Amino acids 1–469). (DOC) [file pone.0038929.s004.doc]

**Table S4. Pairwise identity (% (number of mutations)) in influenza A neuraminidase (NA) protein**

**(Amino acids 1-469**)

|  | **A/Perth/16/2009*** | **A/Victoria/201/2009 X-187*** | **A/Uruguay/716/2007 X-175C*** | **A/Brisbane/10/2007*** | **A/Brisbane/59/2007**** |
| --- | --- | --- | --- | --- | --- |
|  |  |  |  |  |  |
| **A/Victoria/201/2009 X-187*** | 100 () | – | – | – | – |
| **A/Uruguay/716/2007 X-175C*** | 98.7 (6) | 98.7 (6) | – | – | – |
| **A/Brisbane/10/2007*** | 99.4 (3) | 99.4 (3) | 98.9 (5) | – | – |
| **A/Brisbane/59/2007**** | 41.7 (280) | 41.7 (280) | 41.9 (279) | 41.7 (280) | – |
| **A/California/07/2009**** | 43.1 (275) | 43.1 (275) | 43.3 (274) | 43.3 (274) | 81.3 (88) |

* Influenza A/H3N2 subtype

** Influenza A/H1N1 subtype

TIV = trivalent inactivated influenza vaccine

**Note:**

A/Uruguay/716/2007 (NYMC X-175C) was the 2008-09 study TIV component considered antigenically-equivalent to the 2008-09 WHO-recommended H3N2 vaccine strain, A/Brisbane/10/2007. A/Uruguay/716/2007 was used as the “Brisbane-like” 2008-09 H3N2 test antigen in this study.

A/Victoria/201/2009 (NYMC X-187) was the 2010-11 study TIV component considered antigenically-equivalent to the 2010-11 WHO recommended H3N2 vaccine strain, A/Perth/16/2009. A/Perth/16/2009 was used as the 2010-11 H3N2 test antigen in this study.

A/Brisbane/59/2007 was the 2008-09 study TIV H1N1 component as recommended by the WHO, and used as the 2008-09 test antigen in this study.

A/California/07/2009 was the 2010-11 study TIV H1N1 component as recommended by the WHO, and used as the 2010-11 test antigen in this study.

Pairwise identities were calculated from alignments generated with MAFFT (Katoh K, Asimenos G, Toh H (2009). Multiple alignment of DNA sequences with MAFFT. Methods Mol Biol 537:39-64).
